# Supplementary material for: Are carbon emissions trading and green financial instruments synergistic? -Comprehensive quantitative research based on content analysis
Source: PLoS One. 2024 Mar 7;19(3):e0298601. doi: 10.1371/journal.pone.0298601 (PMC10919601; doi:10.1371/journal.pone.0298601)
Supplement: S1 Data — (ZIP) [file pone.0298601.s001.zip › Data/地级市Z_F工作报告.zip!/╦─┤¿/╣π╘¬╩╨/└┤╫╘╣1⁄2╓┌║┼í╛┬φ┐╦╩2╛▌═°í┐_2019.docx]

各位代表：

现在，我代表市人民政府向大会报告工作，请予审议，并请各位政协委员提出意见。

2018年工作回顾

2018年，在省委、省政府和市委的坚强领导下，在市人大、市政协的监督支持下，我们坚定以习近平新时代中国特色社会主义思想为指导，深入学习贯彻党的十九大和习近平总书记对四川工作系列重要指示精神，全面落实省委“一干多支、五区协同”“四向拓展、全域开放”战略部署，坚定实施“三个一、三个三”兴广战略，圆满完成市七届人大三次会议确定的目标任务。全年地区生产总值801.85亿元、增长8.4%，三次产业结构比重由15.4︰44.7︰39.9调整为14.7︰44.7︰40.6，城乡居民人均可支配收入分别达到30592元、11854元，分别增长8.7%、9.7%，增速均居全省第3位，居民消费价格上涨1.6%，地方一般公共预算收入47.69亿元、同口径增长7.3%。

一年来，我们尽锐出战真抓实干，脱贫攻坚再战再捷。始终把提高脱贫质量放在首位，持续打好“四场战役”，圆满完成年度目标任务。青川、昭化、朝天3个贫困县区达到“摘帽”标准，232个贫困村退出、6.25万贫困人口脱贫，贫困发生率下降至1.4%。实施26个扶贫专项，投入财政资金60.71亿元、金融资金36.1亿元、社会资金17亿元。流转城乡建设用地增减挂钩节余指标6800余亩，到位流转资金9亿元。突出抓好稳定增收，贫困人口年人均产业收入达3620元，转移就业8.05万人。易地扶贫搬迁4.94万人。贫困人口、贫困家庭学生全面实现基本医疗、义务教育有保障。大力推动浙广东西部扶贫协作，到位资金1.8亿元，习近平总书记亲自关心的安吉白茶项目落地实施。抓实预警监测和返贫阻击。扎实开展扶贫领域腐败和作风问题专项治理，县级脱贫攻坚项目库建设、脱贫攻坚作风建设“十强十少十不准”等经验在全国全省推广。全省易地扶贫搬迁现场会、推进工业产业扶贫工作会、脱贫攻坚住房安全保障现场会等会议在我市召开。

一年来，我们全力以赴稳定增长，经济运行稳中有进。面对复杂多变的经济形势，全面贯彻落实中央、省稳增长各项决策部署，全市经济加快发展。项目投资持续增长。充分发挥“六大中心”作用，扎实开展项目投资“大比武”，全社会固定资产投资672.21亿元、同口径增长14.1%，增速居全省第4位。民间投资367.46亿元、增长29.4%，增速居全省第4位。集中开工项目6批次，总投资1099亿元、增长17.2%。240个重点项目完成投资449.4亿元、增长14.4%。《广元铁路枢纽总图规划》获批，京昆高速广元至绵阳段扩容前期工作取得突破，绵万高速苍巴段完成招标，上石盘电航枢纽船闸工程、广平高速等交通基础设施项目有力推进，进出川门户型综合交通枢纽加快建设。陵宝二线石龙延伸段、西二环一期、上西互通等基础设施项目竣工投用，康养示范产业园加快推进，广元动车运用所开工建设，主城区城市功能日趋完善。消费潜力不断释放。深入开展市场拓展“三大活动”，培育壮大消费新增长点，社会消费品零售总额405.33亿元、增长11.6%。外贸出口逐步扩大。积极推进外贸综合服务试点，新增外贸企业16家。成功争取设立广元海关，“广元造”产品搭乘中欧班列首发欧洲，阿曼苏丹国广元商品展示馆开馆。外贸进出口总额增长202.2%，出口增长161.9%。要素保障力度加大。强力推进征地拆迁，市本级及市辖区国有建设用地供应总量增长55.2%。强化银行业金融机构支持地方经济发展考核，存贷比提高4.1个百分点，新增小微企业贷款41.43亿元、增长15.6%，新增农业企业贷款9.82亿元、增长11.1%，新增项目贷款327.83亿元、增长13.2%。深入开展财金互动扶贫试点，累计发放贷款3.42亿元。向上争取各类资金235.97亿元、增长13.3%。推进青川县电力供应实现同网同价。实行砂石砖瓦市场限价保供，开展企业用工保障专项行动。

一年来，我们加快产业转型升级，高质量发展取得新成效。工业发展提速增效。聚力发展“6+2”新型工业，全部工业增加值迈上300亿元台阶，规上工业总产值突破1000亿元，战略性新兴产业、军民融合产业占比分别达到18.6%、9.3%。规上工业增加值增长10.8%，增速居全省第5位，上升9位。规上工业利润总额连续23个月增长25%以上。工业增值税增长36.6%，增速居全省第1位。林丰铝电、娃哈哈第二生产基地、劳特巴赫精酿啤酒等支撑性引领性项目落地实施，中国西部绿色家居产业城首期项目入驻开工，工业投资、技改投资同口径分别增长19.9%、11.6%，工业投资增速创近6年来新高。四川省燃气集团在广元注册成立，成为我市首个区域型总部经济实体。新培育规上工业企业50家。新增税收亿元企业1家、2亿元企业1家。夯实产业转移承接平台，新增园区开发面积4500亩、标准化厂房20万平方米。广元经开区成为全国高性能工业铝材产业知名品牌创建示范区。建筑业产值218.74亿元、增长28%，新增施工总承包一级资质企业2家、二级资质企业28家。整合组建广元城投集团和广元建工集团。

现代服务业加快发展。深入实施服务业“三百工程”，服务业增加值325.19亿元、增长9.3%。新培育规上服务业企业40家、限额以上批零住餐企业20家。万贯五金机电城、川北国际汽车城、川陕甘农产品交易中心等项目加快建设，沃尔玛超市入驻广元。举办首届“女皇味道”美食嘉年华活动，地方特色餐饮加快培育。电子商务加快发展，国家级、省级电子商务进农村示范县项目实现全覆盖。中国生态康养旅游名市加快建设，成为全省首批生态康养旅游示范市。黑石坡森林康养旅游度假区等13个重大文旅项目加快推进，新华联曾家山旅游度假区项目成功签约。荣获央视2018《魅力中国城》“魅力城市”称号，唐家河入选“年度魅力生态景区”榜单，纪录片《大蜀道》、广元旅游形象片在央视播出，大蜀道入选全省世界知名线路品牌，嵌入大量广元元素的网络剧《吃货拯救世界》引起较大反响。全年接待游客5028万人次，实现旅游总收入419亿元，分别增长11%、25%，过夜游客数增长22.7%。广元农商行、剑阁农商行挂牌成立，达州银行广元支行开业。年末全金融机构存贷款余额分别增长4.3%、12.5%，金融生态环境评价居全省第5位。房地产市场平稳健康发展，房价保持基本稳定。

现代农业稳步发展。突出抓好“6+7”特色农业产业，农业增加值122.32亿元、增长4.0%，增速居全省第1位。新建现代农业园区8个、村特色产业示范园762个、户办特色产业园7.1万个，新增特色产业基地20.1万亩。苍溪县入选国家现代农业产业园创建单位，全省现代农业园区建设现场推进会在我市召开。粮油持续增产，肉牛羊、剑门关土鸡、生态有机鱼、笋用竹等特色产业加快发展，出栏肉牛增长9.6%、肉羊增长60%、土鸡增长66%，建成省级特色农产品优势区3个。举办广元首届“中国农民丰收节”。发布地方标准8个，新认证绿色农产品和有机产品75个，全国有机产品认证示范市建设加快推进。新增中国驰名商标1个、地理标志证明商标13个、四川农产品优质品牌3个，米仓山茶叶、苍溪红心猕猴桃入围全国区域品牌前100名排行榜，“广元七绝”品牌影响力不断增强。建成农产品初加工处理中心111个，农产品初加工率达55%。新培育国家级农业产业化龙头企业1家、省级7家，国家级示范合作社10家、省级14家，省级示范家庭农场18家，农企利益联结机制覆盖面85%。培育新型职业农民6.8万人。非洲猪瘟、松材线虫病等重大动植物疫病有效防控。

一年来，我们全面深化改革创新和扩大开放，发展潜力不断释放。出台52个专项改革方案，重大改革试点有序推进。创新投资促进体制机制，健全激励约束机制，推进市场化、社会化招商，充分利用中外知名企业四川行、西博会等重大节会平台开展招商活动，引进到位市外资金657.51亿元、增长19.98%。出台优化营商环境20条措施，清理、兑现招商引资优惠政策，落实减税降费措施，降低企业成本20亿元。完善财政金融支农强农机制，“政担银企户”财金互动扶贫模式等经验在全省推广。6家生产经营类事业单位转企改制，组建3家混合所有制企业。国有企事业单位公务用车制度改革基本完成。成功申报全国农村集体产权制度改革整市试点。成立农村土地流转乡镇服务中心230个。创新创业蓬勃发展，新增市场主体2.25万户，返乡人员新创办经济实体3413个；授权专利931件，产出科技成果31项，成果转化产值183亿元；新培育国家级科技型中小企业80家，新增高新技术企业10家，高新技术产业主营业务收入184.5亿元，科技进步对经济增长贡献率达50%。签订区域合作协议16个。举办“5·12”地震10周年纪念和区域合作活动，“九广合作”不断深化，“九地合作”示范区加快建设。成广合作持续深入，承接产业转移项目404个，签约资金491亿元，开工225个，竣工投产106个。协同推进川东北经济区发展，纳入区域“三个十大”的6大项目和平台建设稳步推进。与德国朗茨福特市建立友好城市关系。

一年来，我们坚持融合发展，城乡一体化稳步推进。全市新增建成区面积5平方公里，城镇化率达45.6%。着力优化空间布局，完成市和4个县城市总体规划修编，编制海绵城市等18个专项规划。加快补齐城市基础设施建设短板，严家湾隧道等项目竣工，南山隧道、西二环等项目加快建设。高标准推进三江新区建设，省道205线上石盘至摆宴坝段改线工程等项目竣工投用。新增“百镇建设行动”试点镇15个、省级特色小城镇2个。主城区智慧停车场项目加快建设，新增停车位960个，拥堵问题逐步缓解。城市精细化管理取得新成效。国家园林城市创建有效推进。扎实实施乡村振兴战略，“五个振兴”“十大行动”统筹推进。利州区、五龙镇、青溪镇纳入省级规划试点，启动曾家山等7个市级先行先试试验区。新建幸福美丽新村295个，保护传统村落59个，16个村入选第五批全国传统村落名录。创建省级“四好村”110个。推进农村人居环境整治三年行动，启动建设居民聚居点生活污水处理设施417个，利州区创建为全省农村人居环境整治示范县（区），入库国家贷款项目27个、总投资269.44亿元。创建国家级卫生乡镇3个、省级26个。改造县乡道240公里、通组入户路2400公里，渡改桥13座。新建各类农村供水工程4048处，新增有效灌面2万亩，乐园、双峡湖、曲河3座中型水库大坝主体工程完工。

一年来，我们全力攻坚污染防治，生态优势持续提升。“蓝天保卫战”三年行动首战告捷，城市扬尘、餐饮油烟、露天焚烧、工业排放、机动车尾气污染治理有力有效，市城区环境空气质量优良天数率96.1%，是全省5个达标城市之一，“清新广元”成为常态。河（湖）长制全面落实，城镇污水处理项目加快推进，县级集中式饮用水水源地问题全部整治完成，集中清理白龙湖因洪水输入的垃圾2万余吨，继续实现“一江清水”出广元。青川县、朝天区土壤污染治理和修复项目加快实施，生活垃圾分类稳步推进，畜禽粪污综合利用率和废弃农膜回收率分别达到70%、73%，守住了“一方净土”。接受中央环保督察和省环保督察“回头看”，反馈问题年度整改任务全面完成。城乡环境综合治理深入推进，爱国卫生运动广泛开展。大规模绿化全川广元行动持续推进，完成营造林55.49万亩，森林覆盖率达到56.81%。新建省级森林小镇3个，获评全国最美森林小镇4个。治理水土流失307平方公里。预计单位工业增加值能耗下降6.99%，单位地区生产总值能耗下降4%，主要污染物年度削减目标全面完成。广元经开区成为全省唯一国家级绿色示范园区。

一年来，我们大力发展民生社会事业，人民群众获得感幸福感安全感不断增强。十项民生工程和30件民生实事全面完成，一般公共预算民生支出占比66.6%。城镇新增就业4.1万人，城镇登记失业率3.7%。城乡居民基本养老保险覆盖121.9万人，城乡居民基本医疗保险参保率97%。城乡一体的大病保险、特殊门诊制度进一步完善，社保基金安全完整。农民工工资拖欠问题有效治理。新开工改造危旧房棚户区4755套、基本建成5765套。改造农村危房2.06万户、土坯房5.05万户。新（改）建公厕426座。新增通客车建制村133个，建制村通客车率91.6%。移民库区振兴发展有序推进，移民生产生活条件持续改善。2.5万名农村妇女接受“两癌”免费检查，1万余名农村留守儿童和城乡困境儿童得到关爱保护，11万名残疾人享受到“量体裁衣”式服务。新增养老床位610张。退役军人服务管理得到加强。城市文明程度不断提升，获全国文明城市提名。高质量承办省第十三届运动会、第九届残运会暨第四届特奥会，广元代表团竞赛成绩居全省第2位。新改扩建普惠性幼儿园69所。7个县区义务教育均衡发展全部通过国家评估验收，创建“美丽乡村学校”54所。高中教育质量进一步提升，新增省二级示范高中2所，高考成绩创历史新高，吸引和回流学生2870人。创建国家和省级职业教育示范学校3所，575名往届初高中毕业生返校就读职业学校。市特殊教育学校竣工投用。高等教育稳步发展。公立医院综合改革效果评价居全省第4位，专科专病医疗联合体工作经验在全省推广。分级诊疗制度进一步健全，县域内就诊率达90%。医疗“三监管”实现全覆盖，与域外高端医疗机构合作不断深入，医疗服务质量和水平进一步提升。市县乡村健康信息互联共享，信息化工作经验在全国全省交流。中医药健康产业稳步发展。市县乡村四级公共文化服务体系实现网格化管理，“五馆一站”常态化免费开放，公共文化服务标准化率达75%以上。建成村级农民体育健身工程209个。朝天区被命名为中国民间文化艺术之乡，新增国家级、省级重点文物保护单位20处，一批文艺作品荣获国际、全国、省级奖项。成功应对多年未遇的严重洪涝灾害，无一人员死亡和失踪，修复水毁工程479处，灾后重建全面完成。覆盖城乡的公共法律服务体系建设有力推进，宪法学习宣传和“法律七进”有效开展。认真落实9个防范化解重大风险工作方案，攻坚破解信访突出问题，各类风险有效防控和化解。全面落实安全生产责任制，未发生较大及以上生产安全事故。严格食品药品监管，严厉查处违法案件。深入开展扫黑除恶专项斗争，依法打击各类违法犯罪活动，扎实开展禁毒工作，平安建设群众满意度和社会治安、扫黑除恶、政法干警执法满意度3个单项指标均居全省第1位，再次被评为全国最安全城市。

统计、公积金、国家安全、国防动员、人防、防震、应急管理、气象、老龄、保密、档案、红十字会、双拥、慈善、民族宗教、地方志、科普等工作都取得新成效。

一年来，我们切实加强自身建设，政府职能和作风进一步转变。自觉做到“两个维护”，自觉接受市委领导和市人大监督。“大学习、大讨论、大调研”活动扎实开展。做好接受国务院大督查和省政府大督查工作，被省政府通报表扬。法治政府建设深入推进，首部实体性地方法规《广元市白龙湖亭子湖保护条例》通过实施，重大行政决策五项制度有效落实，法律顾问制度不断完善。大力推进简政放权，清理规范行政权力和公共服务事项并向社会公开，取消行政许可11项，下放委托县区审批事项26项、广元经开区37项。优化审批流程，精简审批要件，推行分阶段并联审批，工程建设项目和广元经开区工业项目审批时限由240个工作日分别压缩至65个、32个工作日以内，投资建设项目审批提速居全省前列。建成政务信息共享交换平台，企业和群众办事提供证明材料减少32%。持续开展“马上办”提速行动，“最多跑一次”事项达到91.8%，居全省第1位。对80岁以上老年人和重度残疾人开展预约上门服务。推进一体化政务服务平台应用，政务服务网办率达95%，居全省第4位。规范涉企行政执法检查，对市场主体检查频次降低75%。招投标领域问题和施工环境整治取得明显成效。建成公共资源交易电子化平台，推进阳光交易。整合61个部门单位服务热线，新建成12345政务服务热线并与省联网试运行。全面推进政务公开，青川县基层政务公开标准化规范化试点工作经验在全国交流。落实全面从严治党和意识形态工作责任，扎实推进党风廉政建设，深入开展“作风纪律深化年”活动，加强审计工作，有效防范各类腐败行为。

各位代表，过去一年取得的成绩，是市委正确决策、坚强领导，团结带领全市人民同心同德、拼搏奋斗的结果，是市人大、市政协有效监督、大力支持的结果。在此，我代表市人民政府，向全市广大干部群众，向人大代表、政协委员，向各民主党派、工商联、无党派人士、各人民团体和社会各界人士，向驻广解放军、武警官兵、消防救援人员、政法干警和民兵预备役人员，向所有关心支持广元发展的朋友们，表示衷心的感谢!

在看到成绩的同时，我们也清醒认识到，我市经济社会发展中还存在一些困难和问题。主要是：产业规模小、结构不优，工业主导地位和带动作用还不够突出。一些重点领域改革效果还未充分体现，开放型经济水平不高。化解政府债务、保障重点项目资金需求办法不多。民营企业融资难融资贵问题尚未有效破解，营商环境仍需优化。保障改善民生任务繁重，脱贫攻坚任务依然艰巨。依法行政水平和政务服务效率还需进一步提高。

2019年主要目标和重点工作

2019年，是我市决战决胜整体连片贫困到同步全面小康跨越的冲刺之年。做好今年政府工作的总体要求是：以习近平新时代中国特色社会主义思想为指导，全面贯彻党的十九大和中央经济工作会议精神，认真落实省委十一届三次、四次全会和省委经济工作会议精神，按照市委七届七次、八次、九次全会和市委经济工作会议部署要求，统筹推进“五位一体”总体布局，协调推进“四个全面”战略布局，坚持稳中求进工作总基调，坚持新发展理念，坚持推动高质量发展，坚持以供给侧结构性改革为主线，坚持深化市场化改革、扩大高水平开放，坚定不移实施“三个一、三个三”兴广战略，全面展开“九项工作大比武”，统筹推进稳增长、促改革、调结构、惠民生、防风险工作，进一步稳就业、稳金融、稳外贸、稳外资、稳投资、稳预期，保持经济持续健康发展和社会大局稳定，为推动整体连片贫困到同步全面小康跨越取得决定性胜利，加快建设川陕甘结合部区域中心城市和四川北向东出桥头堡打下坚实基础，以优异成绩庆祝中华人民共和国成立70周年。

主要预期目标建议：地区生产总值增长8.0%，规上工业增加值增长10.2%以上，全社会固定资产投资增长14.0%以上，社会消费品零售总额增长11.2%以上，地方一般公共预算收入增长与经济增长基本同步，城乡居民人均可支配收入分别增长8.5%以上、9.0%以上，城镇登记失业率控制在4.0%以内，居民消费价格涨幅控制在3.0%以内，城镇化率提高1.5个百分点，完成环境保护和节能减排目标任务。

一、坚决打好三大攻坚战，夯实全面小康基础

强力推进精准脱贫。高质量实现苍溪、旺苍、剑阁3个县“摘帽”、97个贫困村退出、3.2万贫困人口脱贫。完成易地扶贫搬迁任务，实施好农村危房改造和城乡建设用地增减挂钩项目。进一步加强基础设施和公共服务体系建设。落实产业扶贫政策，健全扶贫产品销售体系，大力开展消费扶贫。扎实抓好就业培训，推进扶贫车间建设，拓宽贫困群众就业渠道。加快和规范发展集体经济。落实教育、医疗扶贫政策，强化特殊困难对象兜底保障。深化浙广扶贫协作，创建东西部扶贫协作示范市。继续抓好定点扶贫、驻村帮扶和社会帮扶，做好支持凉山州综合脱贫帮扶工作。认真开展“回头看”“回头帮”，巩固脱贫成果。健全财政投入保障和“四项扶贫基金”稳定补充机制。严格项目资金监管，完善县级脱贫攻坚项目库，落实好项目资金公告公示制度。深入开展扶贫领域腐败和作风问题、惠民惠农财政补贴资金“一卡通”管理问题专项治理。

扎实抓好污染防治。统筹推进山水林田湖草和城乡环境综合治理，继续推进“中国最干净城市”建设，坚决打好污染防治“八大战役”，全面落实河（湖）长制，推进嘉陵江、白龙江上下游协同共治，完成中心城区黑臭水体治理二期工程建设，基本完成城镇污水处理项目，市城区空气质量优良天数率保持在95%以上，主要河流水质达到Ⅱ类，土壤环境质量安全可控。持续抓好生态环境保护和生态文明建设。推进大规模绿化全川广元行动，实施森林质量精准提升、水土保持和生态修复工程。加强生物多样性保护，推进大熊猫国家公园建设。推进新能源示范城市建设、国家低碳城市试点和生活垃圾分类试点，实施餐厨垃圾处理项目。坚持环保与产业发展相协同，实施绿色低碳循环经济“五大行动”，推动绿色生产、绿色生活。搞好第三次国土调查。

防范化解重大风险。坚持底线思维，落实好9个防范化解重大风险工作方案。严格政府债务管理，有效化解存量债务，强化隐性债务管控。严防非法集资，加强互联网金融监管。切实防范化解问题楼盘、特殊群体诉求等重点领域风险。落实安全生产责任制，坚决遏制重特大生产安全事故。加强食品药品安全监管。严防非洲猪瘟等重大动植物疫病。加强应急救援队伍建设，抓好防震、消防、防汛抗旱、应对极端天气和地质灾害防治等工作。

二、牢牢扭住项目投资，推动经济持续快速增长

狠抓项目资金争取。抢抓国家政策窗口期，加强基础设施领域补短板、重大产业、生态环保和公共服务项目谋划储备，确保常态化储备项目1万亿元以上。强化政策研究和资金保障，做深项目前期工作。完善工作机制，统筹整合资源和力量，全方面、多层次向上争取，确保到位项目资金增长10%以上。

加快重点项目建设。强化“六大中心”功能和“四个一”推进机制，开展重点项目集中开工活动。加快推进利州西路棚户区改造、广元智慧旅游综合体、康养示范产业园等项目，实现大唐何家山风电场、川东北储气调峰一期工程等项目竣工，开工建设中石油剑阁天然气净化厂、新华联曾家山旅游度假区、利州凤凰小镇等项目，做好铁路枢纽综合物流基地、青川县城康养旅游综合开发等项目前期工作，确保260个重点项目完成投资450亿元以上。

强化项目要素保障。大力争取地方政府债券资金，扩大企业债发行规模。提升市、县区平台公司信用等级，增强市场化融资能力。推进政府和社会资本合作，鼓励和引导民间投资。完善银行业金融机构支持地方经济发展考核，提高金融机构存贷比，放大担保保证金倍数。出台金融支持制造业发展办法。实行保险业支持地方经济发展考核，推动险资入广。加大征地拆迁力度，加强水电气运、砂石砖瓦、用工等要素保障。

积极扩大消费需求。实施消费升级行动计划，推动实体零售创新转型。持续开展市场拓展“三大活动”和“广元造”产品推介活动，有效提高本地产品市场占有率。开工建设烟波坊餐饮城，启动特色餐饮街区建设和餐饮技能提升工程，打造“女皇味道”特色餐饮品牌。深入实施全企入网、全民触网、电商示范、电商扶贫“四大工程”。积极培育消费热点，发展绿色消费、健康养老家政消费、教育培训托幼消费。开展放心舒心消费创建活动，营造安全放心消费环境。

三、始终坚持高质量发展，加快构建现代产业体系

加速推进新型工业化。突出工业主导地位，做强六大优势特色产业，做大战略性新兴产业和军民融合产业。建立市领导联系指导重点产业工作机制，强化延链招商、补链招商、要素保障等。实现娃哈哈第二生产基地、林丰铝电、劳特巴赫精酿啤酒等重点项目投产，完成工业投资220亿元。做强做大铝产业集群。新增规上企业45家以上。提升产业承载能力，新增园区开发面积3000亩、标准化厂房40万平方米，中国西部绿色家居产业城实现品牌企业入驻。完善促进建筑业发展的政策措施，做大做强建筑业。

大力发展现代服务业。加快现代物流、商贸、金融、电子商务等服务业发展，服务业增加值增长9.0%。加快建设广元动车运用所、万贯五金机电城、川北国际汽车城、川陕甘农产品交易中心等项目，做好广元高铁快运物流基地前期工作。着力培育规上服务业企业。积极培育外贸综合服务企业，发挥中欧班列广元组货基地作用，不断扩大外贸出口。积极引进商业银行，发展非银行金融和金融中介服务机构，完善现代金融服务体系。培育发展房屋租赁市场，引导房地产业转型发展。

加快发展文化旅游经济。推进中心城区“五化”，建设中国生态康养旅游名市核心区，提升旅游城市形象。实施“旅游项目攻坚年”行动，加快大蜀道剑门关旅游区、昭化古城旅游度假区、天曌山、黑石坡森林康养旅游度假区等重点项目建设。支持各县区争创天府旅游名县，推动剑阁县、青川县、朝天区创建国家全域旅游示范区。推进生态、文化、旅游深度融合，加大水上旅游开发力度，推出一批疗休养、研学旅等旅游新产品。加大旅游宣传营销力度，运用互联网、新媒体对主要客源地进行精准化差异化推介，办好女儿节、大蜀道文化旅游节、蜀道马拉松赛等节会活动。强化旅游公共服务体系和配套要素建设，营造优质旅游环境。打造“美丽旅途”，推进旅游标准化建设。力争全年接待游客5500万人次，实现旅游总收入500亿元。

高质量发展现代农业。深入推进农业供给侧结构性改革，重点发展六大特色主导产业，打造七大全产业链集群，因地制宜发展笋用竹、花卉等产业。完成粮食生产功能区和重要农产品生产保护区划定。坚持四级联建、“三园联动”，创建省级现代农业园区2个，建成市级现代农业园区7个，改进和完善既有现代农业园区经营管理。实施农产品品牌建设“五大工程”。大力发展农产品产地初加工和精深加工，积极发展农村电商、休闲农业、乡村旅游等新业态。推进全国有机产品认证示范市建设，建成国家农产品质量安全市。

四、大力实施乡村振兴战略，不断缩小城乡发展差距

全面深化农村改革。深化农村承包地、林地等“三权分置”改革，健全农村产权流转交易市场体系，推动农业适度规模经营。完成全国农村集体产权制度改革整市试点，加大农村集体经济扶持力度。培育壮大农业新型经营主体，扩大农企利益联结机制覆盖。深化供销社综合改革。深化农村投融资改革，推进财政金融支农强农服务创新，推进承包土地经营权抵押贷款试点。鼓励组建县区农业投资公司。深入开展“五到乡”工程，激活农村要素，强化乡村振兴人才、资金、技术支撑。

建设幸福美丽新村。落实“美丽四川·宜居乡村”要求，开展农村人居环境整治“五大行动”，加强路、水、电、气、宽带“五网”建设，建成幸福美丽新村250个。注重保护古村落古建筑，推进产村一体、景村一体。推进“四好农村路”建设，建成省级示范县区1个。健全乡村社会治理体系，建设平安乡村。开展乡村振兴先进县（区）、先进乡镇、示范村省市县“三级联创”。

发展壮大县域经济。出台加快县域经济发展意见，对县域经济发展实行分类考核。坚持“一县一主业”，优化县域产业布局，培育壮大特色优势主导产业，支持县区共建产业链条。争创工业强县、农业强县和生态建设标杆县，争创省级县域经济发展强县、先进县和进步县。推进县域综合改革创新，实施扩权强县、扩权强镇。推进昭化、朝天与中心城区同城化发展，支持苍溪、旺苍、青川建设特色县城和剑阁撤县设市。

五、推进川陕甘结合部区域中心城市和四川北向东出桥头堡建设，不断提升城市功能和品质

加快建设进出川门户型综合交通枢纽。开工建设绵阳至苍溪至巴中高速、京昆高速广元至绵阳段扩容改造、国道212线昭化城区过境段一级公路等项目，加快建设广平高速、广元港进港公路等项目，做好国道542线广元至巴中段一级公路等项目前期工作。持续开展“脱贫奔康交通三年大会战”。推进广巴铁路扩能改造项目前期工作。加快推进张家坝作业区、嘉陵江航道整治和航运配套设施建设，建成上石盘航电枢纽船闸工程。推进广元机场改造项目建设，积极开辟新航线。

聚力建设三江新区。坚持把三江新区作为中心城区发展的主战场，打造高质量发展核心增长极。坚持基础设施建设与产业发展相配套，加快推进宝轮环线（南段）等道路工程，开工建设嘉陵江及白龙江堤防工程、摆宴坝大桥、白龙江大桥、啤酒小镇等项目，实施安全坝、坪雾坝等片区配套基础设施及土地整理等项目，白龙水厂实现投产。实施重大产业项目40个，完成投资70亿元以上。做大做强三江建设投资公司。

推进新型城镇化。加快中心城区、东部新城、北部新城建设，实施南河片区海绵城市、城环南路改造、一环路改造、中央森林康养大道等项目，加快建设南山隧道、北二环东延线、中心城区智慧停车场等项目。实施“城市双修”，创建成国家园林城市，加快公园城市建设。推进“百镇建设行动”，建成试点镇5个、省级特色小城镇1个。加强重点镇规划修编，完善公共基础设施。深化户籍等配套制度改革，加快农业转移人口市民化。推进城市精细化管理，加大违法建设整治力度。

六、积极推进改革创新开放，不断增强发展新动能

深化重点领域改革。实施好已出台改革方案，力争32项国省改革试点早出成果。完善供给侧结构性改革“1+5”方案和配套政策，推动经济转型升级。深化投资促进体制机制改革。落实减税降费政策，清理涉企收费。推进国有企业现代企业制度改革。深化财税体制改革，全面实施预算绩效管理。深化城市执法体制改革。统筹推进行政管理、投融资、社会治理等领域改革。

强化创新驱动。深化厅市会商、市校合作，共建创新平台。积极培育创新主体，争取异地科研成果广元转化，建成铝产业科技成果转化中心。支持科技人员创新创业，引导社会资本参与孵化器、众创空间建设。力争全社会研发投入3.5亿元，高新技术产业主营业务收入200亿元。加强信息基础设施建设，加快发展数字经济，完成川北大数据中心一期工程。推进军民融合。完善科技人才发展体制机制，强化人才支撑。

不断扩大开放合作。落实“四向拓展、全域开放”战略，建设四川内陆开放合作示范市。深化成广合作，新签约项目300个以上。加大承接深圳等地产业转移力度，深化浙广合作，加强与西安等地战略合作。扩大北向开放，大力推介广元食品饮料、农产品、旅游产品。落实川东北经济区“三个十大”建设任务，实施好市际合作协议。支持阆苍南一体化发展。加强川陕革命老区、川陕甘毗邻地区区域合作。拓展与德国、俄罗斯以及日韩、欧美等国际交流合作。充分利用各类重大节会平台，大力开展招商引资，到位市外资金增长15%以上。

大力发展民营经济。全面落实民营经济健康发展政策措施，实现民间投资增长25%以上，民营经济增加值占地区生产总值比重56%以上。健全民营经济工作机制，畅通民营企业反映诉求、解决问题渠道。支持民营企业产品、技术和商业模式创新，推动转型发展。做好小微企业金融服务工作。实施民营企业家素质提升工程，引导民营企业建立现代企业制度，健全法人治理结构。做好第四次全国经济普查工作。

全力提升营商环境。开展“营商环境提升年”行动，系统性优化政务环境、商务环境、法治环境和社会环境。推进简政放权，加快“最多跑一次”改革，全面推行审批服务马上办、网上办、就近办、一次办。推进信息互联互通，助力审批提速。发展连接产业上下游的供应链生产链服务链，完善金融、培训、认证、检验检测等功能性、服务性业态。规范涉企执法检查，全面推行“双随机一公开”联合监管。改进涉企案件办理，减少对企业正常经营活动影响，保护民营企业和企业家合法权益。抓好项目审批服务、招投标、工程建设等领域突出问题整治，持续开展企业和项目建设周边环境治理专项行动。加强诚信政府建设，全面清理兑现招商引资优惠政策，清理拖欠企业账款。规范政商交往，构建“亲”“清”政商关系。加强舆论引导，打造崇商重商文化。

七、加快社会事业发展，不断增进民生福祉

继续办好民生实事。持续加大民生投入，办好重点民生实事。抓好高校毕业生、农民工、退役军人等群体就业，实施家政服务、网络创业、致富带头人“千人培训计划”，确保城镇新增就业3.44万人。强化保障农民工工资支付工作机制。推进全民参保计划和建筑业按项目参加工伤保险，城乡居民基本养老保险覆盖118万人、基本医疗保险参保率97%。深化医保支付方式改革，扩大异地就医联网结算范围，控制医保费用支出。强化社保基金监管。加强城乡养老服务体系建设。改造城镇危旧房棚户区3800套、农村危房4600户、土坯房3.46万户。新增通客车建制村110个，建制村通客车率96%。推进移民库区振兴发展。

发展公平而有质量的教育。深化教育综合改革，加强相关配套建设，稳妥推进高考综合改革。新改扩建普惠性幼儿园38所，开工化解大班额项目学校8所，开展城镇小区配套幼儿园治理，进一步缓解入园难入园贵、大班额问题。巩固提升义务教育均衡发展成果，创建“美丽乡村学校”35所。突出优质高中教育，支持元坝中学、朝天中学创建省二级示范高中。积极发展职业教育，围绕特色优势产业和企业发展需要，深化产教融合、校企合作。积极发展高等教育，支持四川核工业职业技术学院建设。支持和规范社会力量兴办教育。

建设健康广元。深化医药卫生体制改革，建立健全分级诊疗、综合监管、现代医院管理等制度。推进医疗联合体、跨区域专科联盟、重点专科建设，加强与北大医学部、华西医院、常春藤高端医学人才联盟等域外优质资源合作，推进“互联网+健康医疗”，提高医疗服务水平。加强公共卫生工作，推进家庭医生签约服务。巩固提升中医药发展成果。大力发展康养产业，加快建设度众颐康园、市中医院老年疾病楼等重点项目。

繁荣发展文化体育事业。实施“十大文化惠民工程”，优化城乡社区文化服务布局。推进“高清四川·智慧广电”“视听乡村”建设，深化“书香广元·全民阅读”活动。加强文物和非物质文化遗产保护利用，实施武则天历史名人文化传承创新工程，创建国家历史文化名城，建设中子细石器博物馆。发展文化产业，推进影视小镇、女皇文化产业园等项目建设。办好第三届剑门蜀道诗歌节。完善全民健身场地设施，发展竞技体育，推进文体旅融合发展。加强文明细胞建设，推进全国文明城市创建。

做好妇女儿童、青少年、残疾人、老龄、工会、红十字会、慈善、公积金、地方志、档案、科普等工作。

加强和创新社会治理。推进“七五”普法，落实“谁执法谁普法”责任制和法治宣传教育“四项制度”。健全公共法律服务体系。加强基层社会治理，发挥好群团组织作用。推广新时代“枫桥经验”，完善矛盾纠纷多元化解机制。推进信访法治化建设，及时就地解决群众合理合法诉求。深化“平安广元”建设，推进“天网”“雪亮工程”等感知网络建设，完善治安防控体系，深化扫黑除恶专项斗争。抓好维护国家政治安全、维护社会稳定、反恐防暴、禁毒斗争、治安乱点整治等工作。加快市监管中心建设。依法管理宗教事务。加强国家安全教育，提升国防动员能力。做好人民防空、双拥共建、保密、气象等工作。

各位代表！新时代政府工作要有新气象新作为。我们要坚定以习近平新时代中国特色社会主义思想为指导，牢固树立“四个意识”，坚定“四个自信”，坚决做到“两个维护”，全面落实市委决策部署，努力建设法治政府、高效政府、服务型政府、廉洁政府。全面落实法治政府建设纲要，自觉接受市人大及其常委会的法律监督、工作监督和市政协的民主监督，主动接受社会和舆论监督，加强审计监督，抓好行政执法监督，支持司法监督。深入开展调查研究，坚持依法科学民主决策，加强政务公开。坚持依法统计。开展服务能力大提升活动，增强公务人员服务意识，提升服务效率和质量。健全正向激励、容错纠错、考核评价、关心关爱机制，调动广大干部积极性。落实全面从严治党和意识形态工作责任，加强党风廉政建设，持续纠治“四风”。管好用好公共资金，抓好重点领域监管，严防群众身边不正之风和腐败问题。

各位代表！新思想引领新时代，新时代孕育新希望。让我们更加紧密地团结在以习近平同志为核心的党中央周围，在省委、省政府和市委的坚强领导下，奋勇拼搏、团结奋斗，决战决胜整体连片贫困到同步全面小康跨越，加快建设川陕甘结合部区域中心城市和四川北向东出桥头堡，为迎接新中国成立70周年，为推动治蜀兴川广元实践再上新台阶作出新的贡献！
